# Differential Activation of Fetal Hofbauer Cells in Primigravidas Is Associated with Decreased Birth Weight in Symptomatic Placental Malaria

**DOI:** 10.1155/2019/1378174

**Published:** 2019-05-02

**Authors:** Stephanie L. Gaw, Bethann S. Hromatka, Sadiki Ngeleza, Sirirak Buarpung, Nida Ozarslan, Antoinette Tshefu, Susan J. Fisher

**Affiliations:** ^1^Division of Maternal-Fetal Medicine, Department of Obstetrics, Gynecology & Reproductive Sciences, University of California, San Francisco, San Francisco, California, 94143, USA; ^2^Center for Reproductive Sciences, University of California, San Francisco, San Francisco, California, 94143, USA; ^3^Kinshasa School of Public Health, Kinshasa, Democratic Republic of the Congo; ^4^Marmara University, School of Medicine, Istanbul, Turkey

## Abstract

**Background:**

Placental malaria is a leading global cause of low birth weight neonates, especially in first-time mothers. To better understand the role of innate immunity in placental malaria, we investigated the relationships between histopathological markers of placental malaria, fetal and maternal macrophage responses, and perinatal outcomes in a cross-sectional case control study of pregnant women presenting with symptomatic malaria at the time of delivery.

**Results:**

Primigravidas showed increased hemozoin deposition in placental villi (*p*=0.02), syncytiotrophoblasts (*p*=0.01), and fetal Hofbauer cells (*p=*0.01). The percentage of hemozoin-positive villi negatively correlated with infant birth weight (regression coefficient [b] = -0.03 kg decrease in birth weight per % increase in hemozoin-positive villi,* p*=0.035). Malaria-infected placentas showed a twofold increase in Hofbauer cells (*p*<0.001) and maternal macrophages (*p*<0.001). Placental malaria was associated with a threefold increase in the percentage of M2 maternal macrophages (19.2% vs 6.4%,* p*=0.01). Primigravidas showed a significant decrease in the Hofbauer cell M2-percentage in placental malaria (92.7% vs. 97.0%,* p*=0.04), which was predictive of infant birth weight (b=0.08 kg increase in birth weight per % increase in M2 Hofbauer cells,* p*=0.001). There was no association between maternal macrophage response and infant birth weights.

**Conclusions:**

Placentas with malarial infection had increased numbers of fetal Hofbauer cells in the villous stroma and maternal macrophages in the intervillous space. In primigravidas, decreased anti-inflammatory M2-type Hofbauer cells were predictive of lower birth weight. M2-type maternal macrophages were increased in placental malaria, but there was no association with gravidity or birth weight. These results suggested a protective role of M2 Hofbauer cells in fetal growth restriction.

## 1. Background

Each year, approximately 85 million pregnant women worldwide are at risk of* Plasmodium falciparum *malaria infection [1]. Maternal malaria (particularly in primigravidas) leads to poor perinatal outcomes, including low birth weight (BW), fetal growth restriction, and preterm labor [2, 3].

Placental malaria (PM) is characterized by the accumulation of* P. falciparum*-infected red blood cells in the intervillous spaces of the placenta. Deposits of hemozoin (HZ, a malarial by-product) are found in several locations—within the intervillous space, maternal monocytes, the villous cores, and perivillous fibrin deposits [2]. HZ in maternal monocytes circulating within the intervillous space has been consistently associated with low BW [2]. Previously, we found that syncytiotrophoblast denudation was a feature of chronic PM, resulting in direct exposure of the villous cores to maternal blood [4]. Syncytiotrophoblast denudation was associated with villous HZ deposition and maternal monocyte infiltration. HZ stimulates syncytiotrophoblast recruitment of monocytes* in vitro* [5] and triggers other inflammatory pathways [6]. The placental inflammatory response has been associated with poor fetal growth [7–9], possibly through dysfunctional vascularization or nutrient transport [2, 10, 11].

Surprisingly little is known about the role of the fetal Hofbauer cell (HBC), the placental resident macrophage, in PM. HBCs are tissue macrophages localized within the stromal core of the placental villus and are the developing fetus's primary immune cells at the maternal-fetal interface. The functional phenotypes of macrophages are diverse and dynamic, but are broadly classified into two groups: the “classically activated,” M1-type, proinflammatory macrophage and the “alternatively activated,” anti-inflammatory, M2-type macrophage. M1 macrophages form the first line in host defense against a variety of bacteria, protozoa, and viruses, as well as in antitumor immunity. In contrast, the M2 subset is characterized by diverse immunosuppressive activity and includes wound-healing macrophages, IL-10-secreting regulatory macrophages, and tumor-associated macrophages that suppress antitumor immunity. Highly adaptive to their local microenvironment, macrophages have been shown to switch their programming from one functional phenotype to another in response to local signals and are thought to play an important role in maintaining the balance between inflammation (and associated tissue destruction) and the restoration of tissue homeostasis after infection and local injury [12]. Although studies are sparse, HBCs play an anti-inflammatory role in the placenta and may contribute to maternal-fetal tolerance, and most studies have found predominately M2 HBCs in normal placentas [13–15]. Present early in gestation, HBCs are predominately in the M2 anti-inflammatory phenotype and are thought to maintain maternal-fetal tolerance [16–18]. In pathologic states, HBCs are implicated in villitis of unknown etiology, where destructive villous inflammation is associated with poor perinatal outcomes such as preterm labor and intrauterine growth restriction [19].

Here, we characterized the fetal and maternal macrophage responses to PM at the maternal-fetal interface and investigated their association with placental HZ deposits. We also explored the correlations with perinatal outcomes.

## 2. Materials and Methods

### 2.1. Participant Recruitment and Tissue Sampling

The patients and samples in this study were described previously [4]. Briefly, placental biopsies were obtained from singleton deliveries at the Kingasani Maternity Hospital, Kinshasa, Democratic Republic of the Congo. Participants were categorized as malaria-negative controls (n=17) or malaria-positive cases based on the presence of malaria symptoms and peripheral blood smear. Malaria-negative women received antenatal care at the Kingasani Maternity Hospital and intermittent preventative therapy (IPT) per standard of care (two doses of sulfadoxine pyrimethamine for HIV-negative women and three doses for HIV-positive women). Malaria-negative women had no symptoms of malaria upon presentation to the labor and delivery unit, and peripheral smear confirmed the absence of peripheral malaria. Malaria-positive women did not receive antenatal care at Kingasani Maternity Hospital and presented at this institution at the time of delivery with symptoms of malaria; their IPT status was unknown. Malaria-positive women had symptoms such as fever, headache, and joint and muscle pain. Diagnosis of malaria was confirmed with peripheral blood smear test at the time of delivery. Exclusion criteria for this study were common pregnancy complications such as hypertension, preeclampsia, chorioamnionitis, or maternal anemia. The following maternal and neonatal data was collected for all participants: maternal age, gestational age, gravidity, and infant birth weight. Other demographic variables were unknown.

Placental biopsies collected were obtained within 20 minutes of delivery and transferred to 10% neutral buffered formalin (VWR) 10:1 (ml:gram wet weight), made fresh to avoid crystallization artifact. To account for heterogeneity across the maternal-fetal interface, five full-depth biopsies were collected from the center (n=2) and periphery (n=3). Biopsies were fixed for 24 h, transferred to 70% ethanol, paraffin embedded, and sectioned (5 *μ*m).

### 2.2. Immunohistochemistry

For immunohistochemical detection, tissue sections were deparaffinized in xylene and rehydrated in a series of graded ethanol solutions. Antigens were retrieved by heating for 30 minutes at 95°C in 10 mM sodium citrate and 0.05% Tween 20 (pH 6.0). Endogenous peroxidase activity was blocked by incubation in 0.3% H2O2 (30 min at room temperature). Reactivity with mouse anti-cytokeratin-7 (KRT7; OV-TL 12/30, 1:100, Dako) or mouse anti-CD68 (KP1, 9.4 *μ*g/ml, Zymed) identified syncytiotrophoblasts or macrophages, respectively. Binding of primary antibodies (Abs) was detected with biotin-conjugated secondary Abs (Jackson ImmunoResearch) and ABC peroxidase (Vector Laboratories), developed with 3-amino-9-ethylcarbazole (AEC) substrate (Vector Laboratories), and counterstained with hematoxylin. Twelve to 15 randomly chosen fields were scored at 400x for (1) HZ-positive syncytiotrophoblasts, (2) HZ-positive HBCs, and (3) % HZ-positive villi. HZ deposits were confirmed by illumination with polarized light [20].

For double staining experiments, sections were first incubated with mouse anti-CD163 (an M2 marker) (10D6, 1:150, Pierce), followed by biotin-conjugated donkey anti-mouse, ABC peroxidase, and the SG substrate (Vector Laboratories). Sections were washed, reblocked, and incubated with mouse anti-CD68 (a pan-monocyte/macrophage marker) (KP1, 9.4 *μ*g/ml, Zymed). Binding was detected as above and the color developed with AEC substrate. For each sample, six randomly chosen fields representing the entire depth of the biopsy were scored for the number of CD163+/CD68+ (M2) and CD163-/CD68+ (M1) cells in the intervillous (maternal) or intravillous (fetal) compartments. Each sample was evaluated for the number of immunopositive cells of both phenotypes by a blinded scorer on two independent occasions. The percentage of M2 macrophages was calculated as the mean number of (CD163+/CD68+ cells)/(total CD68+ cells) x100.

### 2.3. Statistical Analyses

Data were analyzed with the Welch's t-test, Wilcoxon rank sum test, or logistic regression as appropriate. Histopathologic correlations were determined by Spearman's rank order test with Bonferroni's correction. Linear regression was performed to detect associations with perinatal outcomes. P-values of <0.05 were considered statistically significant. Analyses were performed in Stata 13.

## 3. Results

Although there was a trend toward decreased rates of preterm labor and increased rates of low BW and small for gestational age in patients with malaria, in our cases, PM was not associated with differences in maternal age, gestational age, or infant BW (Table 1). Patients with malaria were less likely to be multigravida (odds ratio [OR] 0.2, confidence interval [CI] 0.0-0.9,* p*=0.04).

**Figure 1:**
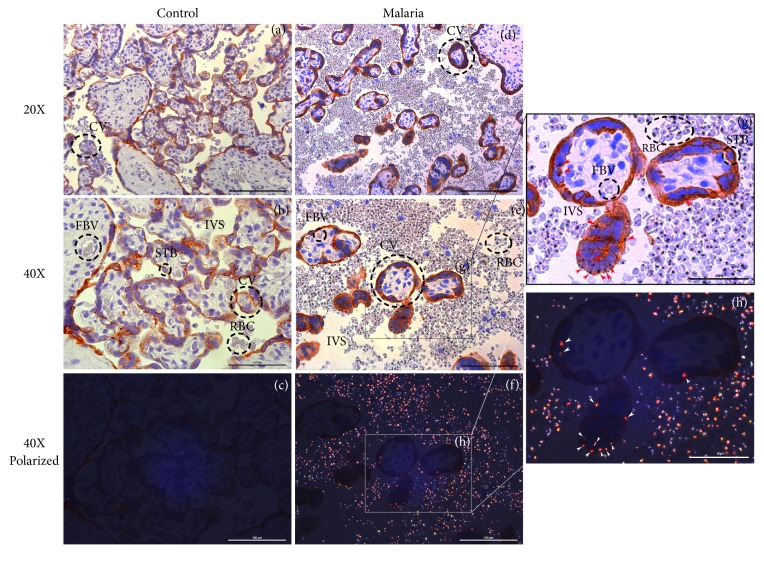
*Hemozoin (HZ) localization within KRT7+ syncytiotrophoblasts of control and placental malaria (PM) samples*. (a)-(c) Control placentas were stained with cytokeratin-7 (KRT7), a trophoblast marker, and evaluated with light microscopy (a, b) and under polarized light filter (c) to assess for the presence of HZ crystals. There was no evidence of* P. falciparum* infection nor HZ in controls. (d)-(h) PM cases stained with KRT7 demonstrated HZ localization within the syncytiotrophoblasts (red arrow in zoom field (g), white arrow in zoom field (h))*. STB*: syncytiotrophoblast,* IVS*: intervillous space,* RBC*: red blood cell,* FBV*: fetal blood vessel.

**Figure 2:**
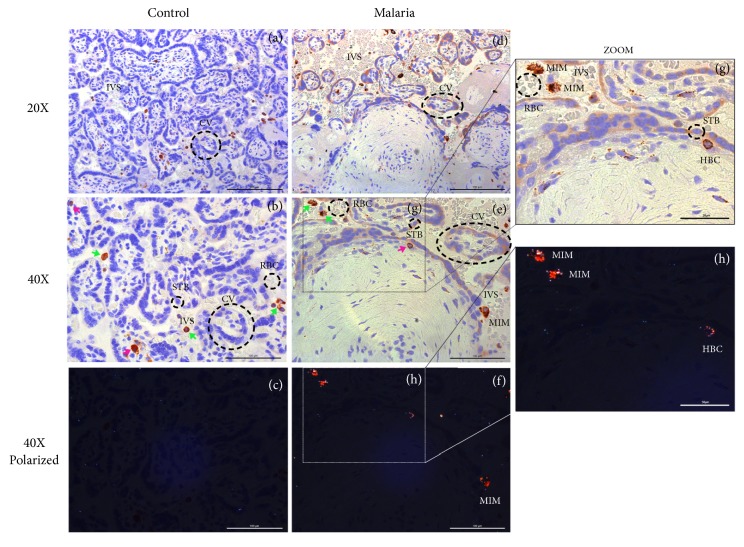
*Hemozoin (HZ) localization within CD68+ Hofbauer cells (HBCs) and maternal intervillous macrophages of control and placental malaria (PM) samples*. (a)-(c) Control placentas were stained with CD68, a monocyte/macrophage marker, and evaluated with light microscopy (a, b) and under polarized light filter (c) to assess for the presence of HZ crystals. There was no evidence of* P. falciparum* infection nor HZ in controls. (d)-(h) PM cases stained with CD68 demonstrated HZ localization within fetal HBCs (in the villous core, pink arrows) and maternal intervillous macrophages (MIM, within the intervillous space, green arrows).* STB*: syncytiotrophoblast,* IVS*: intervillous space,* HBC*: Hofbauer cell,* MIM*: maternal intervillous macrophage.

**Figure 3:**
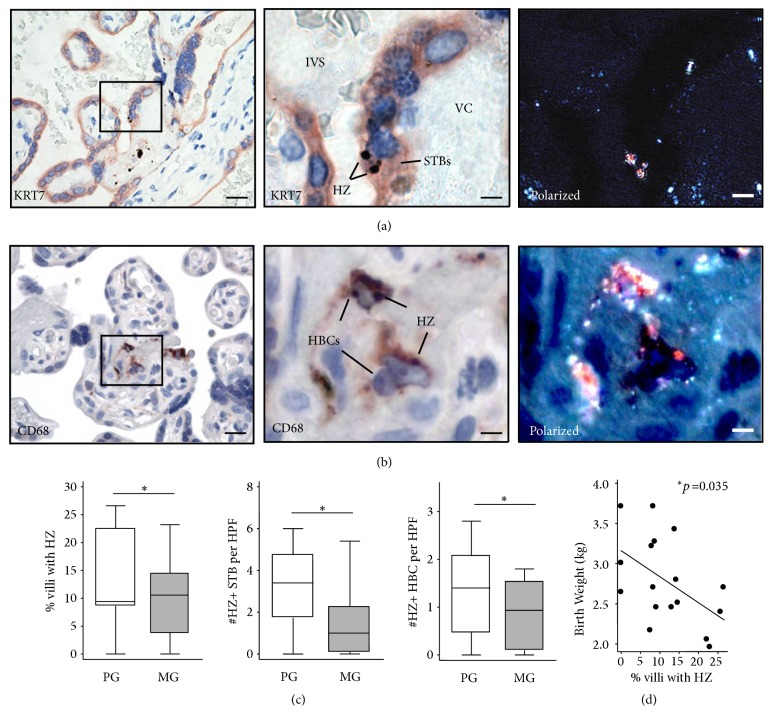
*Hemozoin localization within villous syncytiotrophoblasts and Hofbauer cells (HBCs) and associations with gravidity and birth weight*. (a)-(b) Hemozoin (HZ) colocalized with KRT7+ syncytiotrophoblasts ((a) left with boxed area magnified [middle]) and CD68+ HBCs ((b) left with boxed area magnified [middle]) within the villi of* P. falciparum*-infected placentas. Polarized light enabled the visualization of HZ crystals ((a)* and *(b) right). Bar=40*μ*m (left), 10*μ*m (middle). (c) Primigravidas (PG, white, n=9) showed increased percentage of villi with HZ, numbers of HZ-positive STBs and HZ-positive HBCs, compared to multigravidas (MG, gray, n=8). Data is shown as median, interquartile range, and adjacent values.* P*-values were calculated using Wilcoxon rank sum test. (d) Linear regression showed that birth weight decreased as the percentage of villi containing HZ increased (regression coefficient b= -0.032 kg,* p*=0.035). Abbreviations: KRT7, cytokeratin 7; IVS, intervillous space; VC, villous core; HZ, hemozoin; PG, primigravida; MG, multigravida; STB, syncytiotrophoblast; HBC, Hofbauer cell; HPF, high-powered field.

First, we characterized the HZ-containing villous cells and examined their association with perinatal outcomes. Fetal and maternal macrophages were identified according to their histological location. Experiments that colocalized KRT7 (a trophoblast marker) and CD68 (a pan-macrophage marker) revealed that HZ is found within syncytiotrophoblasts (Figures 1(g)-1(h)), HBCs, and maternal macrophages in PM samples (Figures 2(h)-2(g)). As expected, no HZ was identified in the control placentas (Figures 1(c) and 2(c)). In primigravidas, compared to multigravidas, there were increased percentage of HZ-positive villi (*p*=0.02) and increased numbers of HZ-positive syncytiotrophoblasts (*p*=0.01) and HBCs (*p=*0.01, Figure 3(c)). Linear regression showed a negative correlation between the percentage of HZ-positive villi and infant BW (regression coefficient [b] = -0.03 kg decrease in BW per % increase in HZ-positive villi,* p*=0.035, Figure 3(d)). Correlation analysis also showed a statistically significant association between the percentage of HZ-positive villi and birth weight (Spearman correlation coefficient r= -0.39, p=0.024). No associations were found between HZ localization and gestational age, preterm labor, low BW, or small for gestational age (data not shown).

**Figure 4:**
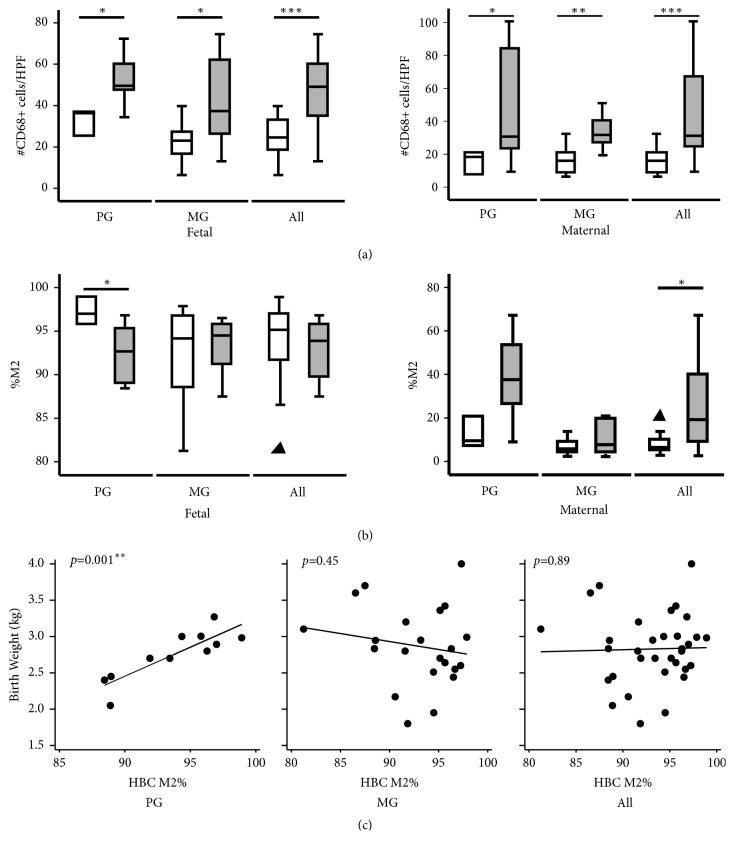
*In primigravidas, activation of fetal macrophages was associated with decreased birth weight*. (a) Increased numbers of fetal CD68+ HBCs (left) and CD68+ maternal macrophages (right) were found in PM (gray) as compared to uninfected controls (white). Upon stratification by gravidity, there were increased HBCs and maternal macrophages irrespective of gravidity. Data is shown as median, interquartile range, and adjacent values.* P*-values were calculated with the Wilcoxon rank sum test. (b) Phenotypic characterization of HBCs (left) and maternal macrophages (right) showed a decrease in HBC M2 percentage in primigravidas with PM, but not multigravidas, as compared to uninfected controls. Overall maternal macrophages showed an increase in M2 polarization with PM. (c) Linear regression analysis showed that the HBC M2 percentage was highly predictive of birth weight in primigravidas, but not multigravidas or all gravidities. Abbreviations: PG, primigravidas; MG, multigravidas; HPF, high-powered field; HBC, Hofbauer cell; PM, placental malaria.

Given that HZ is primarily localized within HBCs, we characterized the macrophage responses in PM. Quantification of fetal CD68+ cells in the intravillous stromal compartment showed a twofold increase in HBC numbers in PM versus uninfected controls (median 49.1 vs. 24.6, respectively,* p*<0.001, Figure 4(a), left panel), suggesting a fetal inflammatory response against PM. A twofold increase in the maternal intervillous macrophage response to PM was found (31.2 vs. 16.0,* p*<0.001, Figure 4(a), right panel). When grouped by gravidity, the increased numbers of HBCs with PM were significant in both primigravidas (49.6 vs. 36.3,* p*=0.03) and multigravidas (1.9-fold, 37.3 vs. 23.0,* p*=0.02). Similarly, the increase in maternal macrophages was significant in both primigravidas (30.7 vs. 18.3,* p*=0.03) and multigravidas (31.7 vs. 16.0,* p*=0.002).

Phenotypic analysis of macrophage polarization toward the proinflammatory M1 or anti-inflammatory M2 state was assessed by calculating the CD163:CD68 ratio [14, 15, 21, 22]. Slides were double-stained with anti-CD163 (an M2 marker) and anti-CD68, and the percentage of M2-type macrophages was calculated. Compared to controls, there was no change in the M2-percentage of fetal HBCs with PM infection (95.5% vs. 93.9%,* p*=0.28, Figure 4(b)). However, there was a threefold increase in the M2-percentage of maternal macrophages (6.4% vs. 19.2%,* p*=0.01, Figure 4(b)). Upon stratification by gravidity, primigravidas showed a significant decrease in the HBC M2-percentage in PM (92.7% vs. 97.0%,* p*=0.04), suggesting an increase in activation. In contrast, maternal macrophages showed a trend toward increased M2-percentage (37.6% vs. 9.5%,* p*=0.05) in the same group. In multigravidas, there was no significant shift in the M2 balance for either fetal (94.5% vs. 94.2%,* p*=0.84) or maternal (7.7% vs. 5.8%,* p*=0.85) macrophages.

Linear regression was performed to determine associations between perinatal outcomes and the numbers and phenotypes of HBCs and maternal macrophages. Increasing M2-percentage in HBCs was highly predictive of BW in primigravidas (b=0.08 kg increase in BW per % increase in M2 HBCs,* p*=0.001, Figure 4(c)), but not in multigravidas (b=-0.02 kg,* p*=0.45), nor with all patients (b=0.003 kg,* p*=0.89). Upon stratification by malaria, we also found a significant association with HBC M2% in primigravidas infected with malaria (b=0.10 kg,* p*=0.004), but not in multigravidas (b= -0.08 kg,* p*=0.31). There were no associations between M2-percentage and BW in maternal macrophages (data not shown).

We next explored potential associations between HZ localization and the fetal and maternal inflammatory responses (numbers and M2-percentages). Correlations were analyzed for PM and gravidity (Table 2). HZ-positive HBCs correlated with the percentage of HZ-positive syncytiotrophoblasts (Spearman's correlation coefficient [*r*]=0.85,* p*<0.001). The maternal macrophage M2-percentage was associated with HZ-positive syncytiotrophoblasts (*r*=0.7,* p*=0.02). Interestingly, the maternal M2-percentage was associated with HZ-positive HBCs (*r*=0.88,* p*=0.03) in primigravidas, but not multigravidas, suggesting that HZ-positive HBCs may influence maternal macrophage polarization in a gravidity-dependent manner. We found no associations between villous HZ localization and HBC numbers nor phenotype. Further, there were no correlations found between numbers of maternal macrophages and HBCs, nor M2-percentages.

## 4. Discussion

In this study, we characterized the cellular localization of HZ in placental villi and assessed the expression of the M2 marker CD163 in association with the fetal and maternal macrophage response in patients with symptomatic placental malaria at the time of delivery. We also investigated the relationships between these histopathologic markers of PM and inflammation, gravidity, and birth outcomes. Poor fetal growth is a hallmark of malaria infection in pregnancy, particularly in first-time mothers. We found a gravidity-dependent HBC response to PM and evidence that a shift in the M2 balance away from the alternative pathway is associated with increased infant BW. In primigravidas, the fetuses had a decrease in anti-inflammatory M2-percentage of HBCs with PM, compared to gravidity-matched controls. Although the phenotypic shift was subtle, it was highly predictive of infant BW, implicating a protective effect of M2 HBCs on fetal growth. This fetal inflammatory response could alter maternal-fetal tolerance and lead to disturbances in placental function. This phenomenon was not observed in multigravidas, perhaps due to partial maternal immunity from prior exposure to PM that decreased exposure to malarial antigens and tempered the overall inflammatory response which can be related to a previous study that described trained innate immunity induced by* P. falciparum* [23]. These findings suggest that fetal inflammation may be a novel mechanism for the increased susceptibility of primigravidas to fetal growth restriction in PM.

Consistent with prior reports [24], we found increased numbers of maternal macrophages during PM infection. Furthermore, our phenotypic analysis showed an increased maternal M2-percentage with PM, but no difference was found when stratified by gravidity, although there was a trend toward increased M2-percentage in primigravidas. There was no correlation between maternal macrophage numbers nor phenotype and clinical outcomes. These results suggested that the fetal HBC response may be a more specific marker of adverse outcomes in this condition. However, the likely complex interactions between maternal and fetal macrophage responses warrant further investigation.

Intriguingly, both fetal and maternal macrophages are strongly recruited to the site of infection, but they were polarized in opposite directions. In the malaria-infected primigravida group, we found decreased M2 polarization of HBCs and a trend toward an increase in M2 maternal macrophages. These findings suggested distinct mechanisms of macrophage stimulation and polarization in fetal versus maternal macrophages. In the setting of decreased fetal anti-inflammatory M2 HBCs against PM, it is possible that the increased maternal M2 response is an attempt to restore the maternal-fetal tolerance that is essential for pregnancy maintenance. Alternatively, the altered fetal M2 response could be that of a naïve immune system reacting to a more significant pathogen challenge in the primigravida. Future studies of the regulatory pathways that are involved in the distinct fetal and maternal innate immune responses may provide additional insights into this hypothesis.

Our study also examined the associations between HZ deposition, gravidity, and perinatal outcomes. We characterized the cellular localization of villous HZ and found an increase in HZ-positive syncytiotrophoblasts and HBCs in primigravidas compared to multigravidas. HZ-positive syncytiotrophoblasts were associated with a maternal M2 response, which is consistent with prior studies [5]. However, there was no association with the fetal macrophage response, suggesting that HBCs are activated through a distinct, yet unknown, pathway.

The primary limitation of our study was the small sample size, which limited our power to detect associations between our histopathologic findings and clinical outcomes. Conclusions regarding preterm labor or fetal growth restriction may be confounded by the lack of confirmed obstetrical dating, which is often a significant limitation of PM studies in endemic settings. Furthermore, the participants with malaria did not receive prenatal care, so other potential contributors to birth outcomes and risk of PM could not be assessed in this study. As such, other pregnancy complications apart from those diagnosed during labor cannot be excluded. Strengths of the study include the prospectively recruited cases and confirmation of active malaria infection by symptoms, peripheral blood smear, and placental histology.

## 5. Conclusions

This study identified a new potential mechanism of poor fetal growth in PM. We found that the fetal HBC response to PM was gravidity-dependent and characterized by a shift in primigravidas of the M2 percentage away from the immune-tolerant M2 phenotype, suggesting activation. This phenotypic shift in HBCs was highly predictive of decreased BW and implicated the fetal inflammatory response as a mediator of susceptibility to poor outcomes in PM.

## Figures and Tables

**Table 1:** Patient characteristics and perinatal outcomes.

	Uninfected	Malaria	*p*
	n=17	n=17	
	Mean ± SD	Mean ± SD	
Maternal age (years)	28.2 ± 6.2	24.5 ± 4.4	0.055
Gestational age (weeks)	37.5 ± 1.5	37.6 ± 1.3	0.807
Birth weight (grams)	2938 ± 464	2771 ± 538	0.340

	n (%)	n (%)	OR (95% CI, *p*)
Gravidity			
Primigravida	3 (18%)	9 (53%)	
Multigravida	14 (82%)	8 (47%)	**0.2 (0.0-0.9, 0.038)**
Preterm labor (<37w)	6 (35%)	3 (18%)	0.4 (0.1-1.9, 0.251)
Low birth weight (<2500g)	1 (6%)	6 (35%)	8.7 (0.9-88.0, 0.059)
Small for gestational age (<10%)	3 (18%)	8 (47%)	4.2 (0.9-19.9, 0.076)

Statistical analysis was performed using Welch's t test (continuous variables) or logistic regression (noncontinuous variables). Confidence intervals that do not overlap the null value of OR=1 are shown in bold.

Abbreviations: SD, standard deviation; RR, relative risk; CI, confidence interval.

**Table 2:** Correlation analysis of hemozoin localization, Hofbauer cell, and maternal intervillous macrophage responses by gravidity.

	HZ+ STB	HZ+ HBC	#HBC	HBC M2%	#MM	MM M2%
*HZ*+* villi*						
Total (n=17)	0.40 *ns*	0.53 *ns*	0.31 *ns*	0.03 *ns*	0.17 *ns*	0.35 *ns*
Primigravida (n=9)	0.42 *p*>0.999	0.55 *p*>0.999	0.12 *p*>0.999	-0.43 *p*>0.999	0.35 *p*>0.999	0.33 *p*>0.999
Multigravida (n=8)	0.22 *p*>0.999	0.45 *p*>0.999	0.34 *p*>0.999	0.34 *p*>0.999	-0.23 *p*>0.999	-0.29 *p*>0.999
*HZ+ STB*						
Total	1	**0.85** ^**∗∗∗**^ **p** < 0.001	0.30 *p=*0.247	-0.18 *p*>0.999	0.20 *p*>0.999	**0.74** **∗** ***p*=0.020**
Primigravida		0.75 *p=*0.419	-0.25 *p*>0.999	-0.52 *p*>0.999	0.07 *p*>0.999	0.82 *p=*0.152
Multigravida		0.88 *p=*0.084	0.42 *p*>0.999	0.04 *p*>0.999	0.36 *p*>0.999	-0.04 *p*>0.999
*HZ+ HBC*						
Total		1	0.29 *p*>0.999	-0.12 *p*>0.999	-0.01 *p*>0.999	0.66 *p=*0.117
Primigravida			0.18 *p*>0.999	-0.45 *p*>0.999	-0.02 *p*>0.999	**0.88** **∗** ***p=*0.033**
Multigravida			0.26 *p*>0.999	-0.07 *p*>0.999	-0.05 *p*>0.999	-0.07 *p*>0.999
#*HBC *						
Total			1	-0.04 *p*>0.999	0.36 *p*>0.999	0.31 *p*>0.999
Primigravida				-0.38 *p*>0.999	0.57 *p*>0.999	-0.07 *p*>0.999
Multigravida				0.32 *p*>0.999	0.14 *p*>0.999	0.46 *p*>0.999
*HBC M2*%						
Total				1	-0.46 *p*>0.999	-0.16 *p*>0.999
Primigravida					-0.57 *p=*0.139	-0.23 *p*>0.999
Multigravida					0.05 *p*>0.999	-0.36 *p*>0.999
#*MM*						
Total					1	0.03 *p*>0.999
Primigravida						-0.23 *p*>0.999
Multigravida						-0.14 *p*>0.999

Spearman's rank correlation coefficients with Bonferroni's correction (*r*) and *p*-values are indicated for each pairwise comparison between % villi with HZ (HZ+ villi), mean numbers of STB with HZ per 400x HPF (HZ+STB), mean numbers of HBC with HZ per 400x HPF (HZ+ HBC), mean numbers of HBC per 400x HPF (#HBC), M2 percentage of HBC (HBC M2%), mean numbers of maternal macrophage (MM) per 400x HPF (#MM), and M2 percentage of MM (MM M2%). Data is shown for the total of all malaria-positive patients (n=17) and stratified by gravidity (primigravidas n=9, multigravidas n=8). Statistically significant values, defined as *p*<0.05, are shown in bold.

Abbreviations: HZ, hemozoin; STB, syncytiotrophoblast; HBC, Hofbauer cell; MM, maternal macrophage; HPF, high-powered field

## Data Availability

The data used to support the findings of this study are available from the corresponding author upon request.

## Abbreviations

BW: Birth weight
CI: Confidence interval
HBC: Hofbauer cell
HPF: High-powered field
HZ: Hemozoin
IVS: Intervillous space
KRT7: Cytokeratin 7
MG: Multigravida
OR: Odds ratio
PG: Primigravida
PM: Placental malaria
STB: Syncytiotrophoblast
VC: Villous core.

## References

[1] Dellicour S., Tatem A. J., Guerra C. A., Snow R. W., ter Kuile F. O. (2010). Quantifying the number of pregnancies at risk of malaria in 2007: a demographic study. *PLoS Medicine*.

[2] Umbers A. J., Aitken E. H., Rogerson S. J. (2011). Malaria in pregnancy: small babies, big problem. *Trends in Parasitology*.

[3] Dimasuay K. G., Aitken E. H., Rosario F. (2017). Inhibition of placental mTOR signaling provides a link between placental malaria and reduced birthweight. *BMC Medicine*.

[4] Hromatka Bethann S. B. S., Ngeleza S., Adibi J. J., Niles R. K., Tshefu A. K., Fisher S. J. (2013). Histopathologies, immunolocalization, and a glycan binding screen provide insights into plasmodium falciparum interactions with the human placenta. *Biology of Reproduction*.

[5] Lucchi N. W., Sarr D., Owino S. O., Mwalimu S. M., Peterson D. S., Moore J. M. (2011). Natural hemozoin stimulates syncytiotrophoblast to secrete chemokines and recruit peripheral blood mononuclear cells. *Placenta*.

[6] Olivier M., Van Den Ham K., Shio M. T., Kassa F. A., Fougeray S. (2014). Malarial pigment hemozoin and the innate inflammatory response. *Frontiers in Immunology*.

[7] Kabyemela E. R., Fried M., Kurtis J. D., Mutabingwa T. K., Duffy P. E. (2008). Fetal responses during placental malaria modify the risk of low birth weight. *Infection and Immunity*.

[8] Conroy A. L., Silver K. L., Zhong K. (2013). Complement activation and the resulting placental vascular insufficiency drives fetal growth restriction associated with placental malaria. *Cell Host & Microbe*.

[9] Chandrasiri U. P., Chua C. L., Umbers A. J. (2014). Insight into the pathogenesis of fetal growth restriction in placental malaria: decreased placental glucose transporter isoform 1 expression. *The Journal of Infectious Diseases*.

[10] Boeuf P., Aitken E. H., Chandrasiri U. (2013). Plasmodium falciparum malaria elicits inflammatory responses that dysregulate placental amino acid transport. *PLoS Pathogens*.

[11] Umbers A. J., Boeuf P., Clapham C. (2011). Placental malaria-associated inflammation disturbs the insulin-like growth factor axis of fetal growth regulation. *The Journal of Infectious Diseases*.

[12] Murray P. J., Wynn T. A. (2011). Protective and pathogenic functions of macrophage subsets. *Nature Reviews Immunology*.

[13] Tang Z., Abrahams V. M., Mor G., Guller S. (2011). Placental Hofbauer cells and complications of pregnancy. *Annals of the New York Academy of Sciences*.

[14] Kim S. Y., Romero R., Tarca A. L. (2012). Methylome of fetal and maternal monocytes and macrophages at the feto-maternal interface. *American Journal of Reproductive Immunology*.

[15] Yang S. W., Cho E. H., Choi S. Y. (2017). DC-SIGN expression in Hofbauer cells may play an important role in immune tolerance in fetal chorionic villi during the development of preeclampsia. *Journal of Reproductive Immunology*.

[16] Sisino G., Bouckenooghe T., Aurientis S., Fontaine P., Storme L., Vambergue A. (2013). Diabetes during pregnancy influences Hofbauer cells, a subtype of placental macrophages, to acquire a pro-inflammatory phenotype. *Biochim Biophys Acta*.

[17] Joerink M., Rindsjö E., Van Riel B., Alm J., Papadogiannakis N. (2011). Placental macrophage (Hofbauer cell) polarization is independent of maternal allergen-sensitization and presence of chorioamnionitis. *Placenta*.

[18] Nagamatsu T., Schust D. J. (2010). The contribution of macrophages to normal and pathological pregnancies. *American Journal of Reproductive Immunology*.

[19] Kim J.-S., Romero R., Kim M. R. (2008). Involvement of Hofbauer cells and maternal T cells in villitis of unknown aetiology. *Histopathology*.

[20] Romagosa C., Menendez C., Ismail M. R. (2004). Polarisation microscopy increases the sensitivity of hemozoin and Plasmodium detection in the histological assessment of placental malaria. *Acta Tropica*.

[21] Cornelissen R., Lievense L. A., Maat A. P. (2014). Ratio of intratumoral macrophage phenotypes is a prognostic factor in epithelioid malignant pleural mesothelioma. *PLoS ONE*.

[22] Takeuchi H., Tanaka M., Tanaka A., Tsunemi A., Yamamoto H. (2016). Predominance of M2-polarized macrophages in bladder cancer affects angiogenesis, tumor grade and invasiveness. *Oncology Letters*.

[23] Schrum J. E., Crabtree J. N., Dobbs K. R. (2018). Cutting edge: Plasmodium falciparum induces trained innate immunity. *The Journal of Immunology*.

[24] Rogerson S. J., Hviid L., Duffy P. E., Leke R. F., Taylor D. W. (2007). Malaria in pregnancy: pathogenesis and immunity. *The Lancet Infectious Diseases*.

